# Public health priorities for Sino-Africa cooperation in Eastern Africa in context of flooding and malaria burden in Children: a tridecadal retrospective analysis

**DOI:** 10.1186/s12889-023-16220-7

**Published:** 2023-07-11

**Authors:** Joseph Kimuli Balikuddembe, Jan D. Reinhardt, Wen Zeng, Habteyes Tola, Baofeng Di

**Affiliations:** 1 Institute for Disaster Management and Reconstruction, Sichuan University and Hong Kong Polytechnic University, Chengdu, Sichuan China; 2 East African Center for Disaster Health and Humanitarian Research, Kampala, Uganda; 3 Swiss Paraplegic Research, Nottwi, Switzerland; 4 Department of Health Sciences and Medicine, University of Lucerne, Lucerne, Switzerland; 5 Rehabilitation Medicine Center, The first Affiliated Hospital of Nanjing Medical University, Nanjing, China; 6 Department of Public Health, College of Health Sciences, Salale University, Fiche, Ethiopia; 7 Center for Archaeological Science, Sichuan University, Chengdu, Sichuan China

**Keywords:** Flooding, Malaria, Forum for China-Africa Cooperation (FOCAC), Eastern Africa, Public health

## Abstract

**Background:**

Malaria remains a major public health burden to children under five, especially in Eastern Africa (E.A), —a region that is also witnessing the increasing occurrence of floods and extreme climate change. The present study, therefore, explored the trends in floods, as well as the association of their occurrence and duration with the malaria incidence in children < 5 years in five E.A partner countries of Forum for China-Africa Cooperation (FOCAC), including Ethiopia, Kenya, Somalia, Sudan, and Tanzania between 1990 and 2019.

**Methods:**

A retrospective analysis of data retrieved from two global sources was performed: the Emergency Events Database (EM-DAT) and the Global Burden of Diseases Study (GBD) between 1990 and 2019. Using SPSS 20.0, a correlation was determined based on *ρ*= -1 to + 1, as well as the statistical significance of *P* = < 0.05. Time plots of trends in flooding and malaria incidence were generated in 3 different decades using R version 4.0.

**Results:**

Between 1990 and 2019, the occurrence and duration of floods among the five E.A partner countries of FOCAC increased and showed an upward trend. On the contrary, however, this had an inverse and negative, as well as a weak correlation on the malaria incidence in children under five years. Only Kenya, among the five countries, showed a perfect negative correction of malaria incidence in children under five with flood occurrence (*ρ =* -0.586**, *P-value =* 0.001) and duration (*ρ =* -0.657**, *P-value = <* 0.0001).

**Conclusions:**

This study highlights the need for further research to comprehensively explore how different climate extreme events, which oftentimes complement floods, might be influencing the risk of malaria in children under five in five E.A malaria-endemic partner countries of FOCAC. Similarly, it ought to consider investigating the influence of other attributes apart from flood occurrence and duration, which also compound floods like displacement, malnutrition, and water, sanitation and hygiene on the risk and distribution of malaria and other climate-sensitive diseases.

## Introduction

Malaria remains a major public health burden significantly contributing to morbidity and mortality, especially in children under five years of age in the highlands, tropical, and sub-tropical parts of the world [1–3]. It is an infectious febrile disease transmitted to the population through the bites of infected female Anopheles mosquitoes. Some of the clinical symptoms of malaria presented include fever, chills, body aches, headache, cough, diarrhea, muscle or joint aches, malaise, loss of appetite, nausea and vomiting [2, 4]. Although malaria is preventable and curable, it is mostly affecting under-five children who account for 61% of all malaria deaths worldwide. On average, for example, over 600,000 African children under the age of five die from malaria each year [1]. Between 2021 and 2022, the global prevalence of malaria was reported at 247 million, according to the recent World Malaria Report [1]. The disease is also associated with measurable direct and indirect economic costs, annually estimated to impose a loss of over 1.3% on the gross domestic product (GDP) of the most affected countries, as a result of the treatment costs and time lost for work [5, 6]. Malaria represents about 13% of all infectious diseases and 90% of its associated deaths are reported to be happening in Sub-Saharan Africa (SSA), covering also the Eastern Africa (E.A) region [1, 7]. Although wide-scale control and prevention interventions, which largely involve sleeping under mosquito nets, antimalaria medicines, and indoor residual spraying of insecticides, have been undertaken; malaria is still endemic in E.A [1, 2, 8, 9].

The impact of climate change and its associated extreme events like flooding on the incidence of vector-borne diseases (VBDs), including malaria, has been of great concern for decades [3, 10–12]. Out of 432 total disasters that were registered in 2021, floods dominated with 223 (∼50%) events, up from 163 annual events that were recorded to have occurred previously in 2020 [13]. Floods are reported to increasingly affect less-resourced and developing regions like E.A [14–18]. It has also been previously reported that climate change and its extreme events beyond floods are imposing a heavy brunt of malaria transmission on countries in E.A and SSA [1, 3, 7, 19–23]. In spite of this, there are still insufficient studies elucidating how the burden of malaria in the context of flooding impacts some of the vulnerable groups like children who are often in the greatest need but receive little attention during disasters and public health emergencies [24].

In recent years, China has made substantial commitments to assist Africa to overcome a wide range of challenges through the Forum for China-Africa Cooperation (FOCAC), also referred to as the Sino-Africa Cooperation [25, 26]. To date, however, there’s little attention or barely no attention paid to investigating how the FOCAC initiative can reinvigorate flood risk management (FRM) and malaria amelioration. This is particularly so with E.A where, as aforementioned, malaria is still endemic and killing over half a million children [1, 2, 8, 9]. In light of this, the present study explored the trends in floods and the association of their occurrence and duration with malaria incidence in under-five children in five E.A partner countries of FOCAC, including Ethiopia, Kenya, Somalia, Sudan, and Tanzania between 1990 and 2019. At last, this study identified how some of the promises in the agenda of FOCAC in line with public health can be prioritized in addressing the malaria burden among the five E.A countries.

## Methods and materials

### Study design

A 30-year retrospective analysis of data from two global sources was performed: the Emergency Events Database (EM-DAT) and the Global Burden of Diseases Study (GBD) between 1990 and 2019. Retrospective data analysis was considered because it is appropriate and routinely used to retrieve data from either the entire source of data or its subset to help to answer the key research questions of interest [27].

### Study area

The E.A region, which is located in SSA, comprises among other countries Ethiopia, Kenya, Somalia, Sudan, and Tanzania, which have been focused on in this study. The E.A was considered because it is one of the most fragile regions across the globe having countries encountering various political and socioeconomic challenges such as conflicts and wars, displacement and refugees, infectious diseases, poverty, famine, and population growth [16, 17, 28–31]. Most of the E.A countries share topographical, geomorphological, and meteorological features, environment and ecology, and natural hazards. Moreover, the five countries above were selected from the E.A region since they were reported among the 7 flood-prone countries in Africa in a study by Li et al. [32].

### Data sources

*a) EM-DAT* The Emergency Disasters Database (EM-DAT) is maintained by the Centre for Research on the Epidemiology of Disasters (CRED) of the Université Catholique de Louvain in Brussels, Belgium [33]. EM-DAT is a freely available global database and contains to date data on more than 22,000 disasters worldwide attributable to natural, man-made or technological and complex hazards from 1900 to the time of writing. Disaster classification in EM-DAT is based on and adapted from the Integrated Research on Disaster Risk Peril Classification and Hazard Glossary [34]. For an event to be included in the EM-DAT, it must meet at least one of the following: (1) 10 or more fatalities; (2) 100 or more affected individuals; (3) a declaration of a state of emergency; or (4) a call for international assistance, for instance, from sources as United Nations (UN) agencies, governmental and non-governmental agencies (NGO), insurance companies, research agencies, and press agencies [33]. A detailed description of EM-DAT including its structure, classification, variables, and definitions is available here: https://www.emdat.be/guidelines.

EM-DAT data are collated from reliable sources such as the UN and NGOs, and they are assembled based on over 50 variables among others including, country, region, disaster number, year, occurrence dates (the start and end day(s), month(s), and year(s)), disaster group, disaster sub-group, disaster type, disaster sub-type, disaster sub-sub-type, number of injured, event name, total deaths, number of affected, number of homeless, total affected population, and total economic damages (adjusted in ‘000’ USD) [33].

#### b) GBD

Data about the incidence of malaria in children under five were extracted from the GBD study, which is coordinated by the Institute for Health Metrics and Evaluation at the University of Washington, USA [35]. GBD consists of systematic and scientific data quantifying the comparative magnitude of health losses due to diseases, injuries, and risk factors by sex, age, and location over time, aimed at enhancing healthcare systems and eliminating health disparities among 204 countries and territories [36, 37]. We chose GBD because it is a global open-access database that allows users to explore its data at the national, regional and international levels —spanning over three decades between 1990 and 2019. To achieve the aim of this study, we deemed it appropriate to only consider data on malaria incidence in children under-five years other than that on their prevalence or mortality. In this case, the data on malaria incidence in general that is contained in the GBD can help to support the diverse needs, in particular, by informing the policy and research-related interventions for malaria surveillance and control in the context of flooding and other hazards of climate change.

### Data extraction

#### a) EM-DAT

This database was accessed on numerous occasions between 1st and 30th September 2022 by one author (JBK) using his registered username and password to retrieve data about recorded floods in countries considered in this study from 1990 to 2019. This period was assumed to be appropriate in analyzing the occurrence of floods and their impacts among the five E.A countries so that in the future it can help to inform the relevant strategies for flood mitigation. Firstly, the following search parameters were applied to query the database: disaster group: ‘natural’; disaster subgroup: ‘hydrological’ disaster type: ‘flood’; location: continent ‘Africa’; region: ‘Eastern Africa’; period or from: ‘1990–2019’. Second, flood data meeting these parameters were downloaded from the EM-DAT in comma-separated values (CSV) format —which facilitates the processing of data in tabular format. Third, the data were manually checked for plausibility and then sorted in ascending order by the variable “country”. Thereafter, the data for five E.A countries under the review period (1990–2019) were considered for detailed analysis. It should be noted that any missing data were not imputed.

#### b) GBD

Data about the incidence of malaria in GBD for five E.A countries was retrieved in line with the current International Classification of Diseases and Injuries (ICD-11) [38]. This aimed at exploring the potential influence of flood occurrences and duration on malaria incidence in under-five children in five E.A countries. GBD was accessed via the Global Health Data Exchange website https://www.healthdata.org/gbd/2019 [35] by filtering and extracting its annual data between 1990 and 2019 on the incidence rate of malaria among under-five children (age-standardized of all ages and measured by case per 100,000). As noted before, children of this age bracket were considered because malaria is reported to be the leading cause of their morbidity and mortality in endemic settings [1]. Like with EM-DAT, the author (JBK) had to access and retrieve data using his registered account in the GBD. Thereafter, the data from 1990 to 2019 for each E.A country considered herein was retrieved from Microsoft Excel Spreadsheet for analysis.

### Data analysis

Statistical Package for the Social Sciences (SPSS) version 20.0 (International Business Machines Corporation) was used for statistical analysis. At first, the duration for each flood event was calculated in terms of the days it took to occur between the start and end dates. Thereafter, the occurrence duration and three other variables, including the year, country, and number of occurrences among the five E.A countries, were used to calculate and generate the percentages and graphs in the Microsoft Office Excel version 2019 (Microsoft Corporation, Redmond, WA, USA) following the standard methods of descriptive statistics.

Using SPSS, the mean differences at 95% confidence intervals (CIs), standard deviations (Std Dev), correlation coefficient (*ρ*), and statistical significance (*P-values*) were conducted. Numerical bivariate analysis based on the Spearman rank-order correlation test (Spearman-rho) was used to establish the potential influence of flood occurrence and duration on malaria incidence in children < 5 years in five partner countries of FOCAC in E.A between 1990 and 2019. The *ρ* was evaluated based on -1 to + 1, whereby, 0 indicates that the two variables are uncorrelated; +1 is a positive correlation; and − 1 is a negative correlation. In this case, the larger value represents a stronger correlation. The statistical significance of *P = <* 0.05 was identified to be statistically significant.

### Trends plotting

R version 4.0 with the ggplot2 package (R Development Core Team, Vienna, Austria) was used to generate polylines for plotting and visualizing the trends in malaria incidence in children under five, and on the other hand, flood occurrences in three separate decades (1990–1999, 2000–2009, and 2010–2019) among five partner countries of FOCAC in E.A. R is a free software environment for statistical computing and graphics [39].

### Ethics approval

This study required no ethics approval since it did not directly involve humans.

## Results

**Fig. 1:**
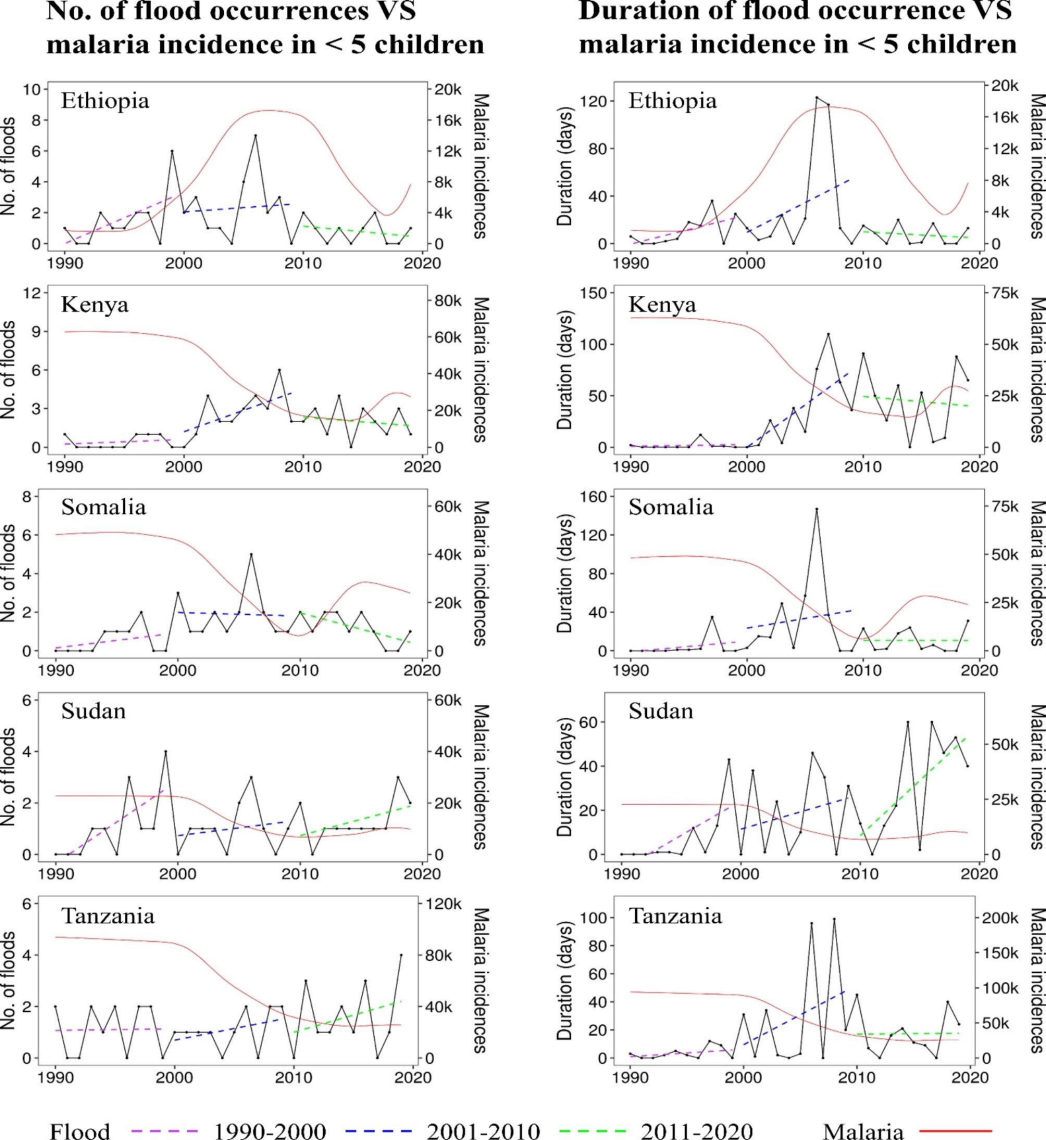
Trends in flood occurrences and duration compared with malaria incidences in children < 5 years among the five partner countries of FOCAC in E.A between 1990 and 2019

*Trends in flood occurrences*: A total of 205 floods were retrieved in EM-DAT to have occurred in five partner countries of FOCAC in E.A within thirty years. They were distributed as follows: Kenya 51(24.9%), Ethiopia 46(22.4%), Tanzania 38(18.5%), Somalia 36(17.6%), and Sudan 34(16.6%). According to Table 1, Ethiopia and Kenya registered the largest number of floods, which on average occurred 7 and 6 times respectively, annually. In the 1st decade (1990–1999), based on Fig. 1, Ethiopia, and Sudan registered upward trends in floods whereas Kenya, Somalia, and Tanzania had their trend curves flattened. In the 2nd decade (2000–2009), interestingly, the trend in floods in Kenya this time round sharply went upwards, unlike in the remaining four countries where their trends in flood occurrences remained relatively flat. In the last decade (2011–2019), trends in flood occurrences in Ethiopia, Kenya, and Somalia declined, unlike in Sudan and Tanzania, which recorded a slight increase in flood occurrences. It can be observed that the occurrences of floods varied among five E.A countries in three decades, and in most cases, their trends climbed upwards. Inevitably, this could have been aggravated by some risk factors for flooding such as the prolonged rainfall or population encroachments and settlements in the flood-prone areas, which happened in different years within the three decades.

### Trends in the duration of flood occurrences

**Fig. 2:**
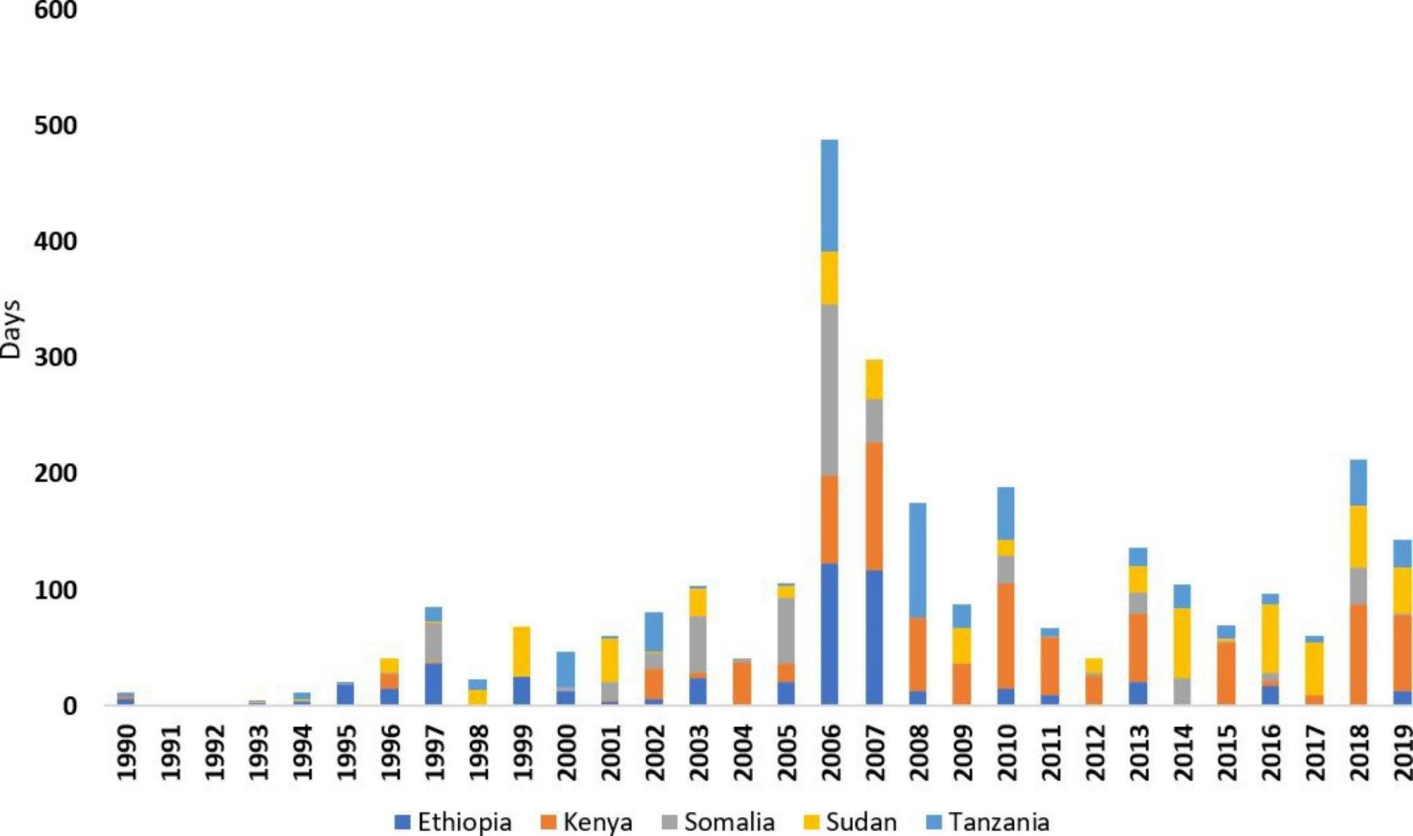
The duration (days) of flood occurrence per year across the five partner countries of FOCAC in E.A, 1990–2019

The occurrence of 205 floods happened within a total of 2,869 days in five partner countries of FOCAC in E.A between 1990 and 2019. The highest number of days 833(29%) was recorded in Kenya and followed by Sudan, Ethiopia, Tanzania, and Somalia with 566(19.7%), 501(17.5%), 497(17.3%), and 472(16.5%), respectively (Fig. 2). Based on data from EM-DATA, the longest flooding event lasted for 147 days in 2006 in Somalia, as well as 117 days in 2007 in Ethiopia. Similar to trends in the first decade, floods that were recorded with a notable increase in the duration of occurrence happened in Ethiopia and Sudan compared to Somalia and Tanzania (with slightly lower trends) and Kenya (with flat trends). In the second decade, all five E.A countries had their trends in duration of flood occurrences going upwards, especially in Kenya and Tanzania. This could be attributed to the evolution of floods and other hydrometeorological hazards that rapidly manifested and were possibly exacerbated by climate change. In the third decade, trends in the duration of flood occurrences rose only in Sudan. On the other hand, Ethiopia and Kenya had their trends in the duration of flood occurrences substantially falling compared to Somalia and Tanzania where they moderately flattened.

*Trends in malaria incidence in children under five years*: According to Table 1, the average incidence rates of malaria in children were annually distributed as follows: Tanzania 59,876.9 (38.83%); Kenya 39,846.3 (25.84%); Somalia 32,003.6 (20.75%), Sudan 14,949.2 (9.69%); and Ethiopia 7,522.6 (4.87%). Apart from Ethiopia —where trends in incidence of malaria were low in the first decade based on Fig. 1, the trends in the remaining four countries prevailed upwards. Again, aside from Ethiopia, which had its trends spiking in the second decade, the incidence of malaria in four other countries suddenly declined. In the last decade, both rising and declining trends in malaria incidence were registered in four countries except Tanzania, where they have continuously declined since the previous decade. The declines in trends of malaria incidence, especially in the 1st and 2nd decades, can be associated with the interventions that were zealously scaled-up by the government and non-governmental actors to control and ameliorate the burden of malaria among the five partner countries of FOCAC in E.A.

### Correlation of flood occurrence and duration with malaria incidence in children under five years

According to Table 1, the rate of malaria incidence in children < 5 years showed a moderately significant, albeit weak and negative correlation with flood occurrences within the three decades. This was only observed in Kenya (*ρ* = − 0.586**, *p-value* 0.001) and Somalia (*ρ =* − 0.421**, *p-value* = 0.021). In the remaining three E.A countries, including Ethiopia (*ρ =* 0.264, *p-value* = 0.165), Sudan (*ρ =* -0.207, *p-value* = 0.272), and Tanzania (*ρ =* -0.184, *p-value* = 0.331); there was an insignificant correlation of flooding with the incidence of malaria in children under five years between 1990 and 2019. On the other hand, apart from Somalia (*ρ =* -0.268, *p-value* = 0.153), the duration of flood occurrences was revealed to be statistically significant and associated with malaria incidence in under-five children all over the study period. Based on Table 1, this was registered as follows: Ethiopia (*ρ* = 0.294*, *p-value* = 0.0317), Kenya (*ρ =* − 0.6503**, *p-value* = < 0.0001), Sudan (*ρ =* − 0.396*, *p-value* = 0.033), and Tanzania (*ρ =* − 0.459**, *p-value* = 0.011). In this case, only Kenya, among the five countries, showed a perfect negative association with malaria incidence in children under five years of age and flood occurrence and duration. All these results indicate inverse and negative, as well as weak correlations of flood occurrence and duration with the incidence of malaria in children < 5 years in five partner countries of FOCAC in E.A.

**Table 1:** Correlation of flood occurrence and duration with the malaria incidence in children < 5 years in five partner countries of FOCAC in E.A, 1990–2019

		Incidence in children < 5 years	No of Occurrence	Correlation Coefficient Sig. (2-tailed)	Duration of Occurrence	Correlation Coefficient Sig. (2-tailed)
**Ethiopia**	Minimum	1779	0		0	***ρ =*** 0.294*
	Maximum	14670.1	7	*ρ =* 0.264	123.0	***P-value =*** **0.0317**
	Mean	7522.6	1.53	*P-value =* 0.159	16.7	
	Std Dev	4852.1	1.717		29.7	
**Kenya**	Minimum	14996.2	0	***ρ =*** -0.586**	0.0	***ρ =*** -0.657**
	Maximum	62888.8	6	***P-value =*** **0.001**	110.0	***P-value = <*** **0.0001**
	Mean	39846.3	1.7		27.8	
	Std Dev	19521.1	1.557		33.5	
**Somalia**	Minimum	6090.6	0	***ρ =*** − 0.421**	0	*ρ =* -0.302
	Maximum	49031.2	5	***P-value =*** **0.021**	147.0	*P-value = 1.039*
	Mean	32003.6	1.2		15.7	
	Std Dev	15006.8	1.095		29.5	
**Sudan**	Minimum	6746.6	0	*ρ =* -0.205	0.0	***ρ =*** -0.420*
	Maximum	22667.7	4	*P-value =* 0.278	60.0	***P-value =*** **0.021**
	Mean	14949.2	1.13		18.9	
	Std Dev	6821.9	1.042		20.8	
**Tanzania**	Minimum	24594.8	0	*ρ =* -0.184	0.0	***ρ =*** − 0.457**
	Maximum	93882.1	4	*P-value =* 0.331	99.0	***P-value =*** **0.011**
	Mean	59876.9	1.27		16.6	
	Std Dev	29348.5	1.048		25.4	

Note: *Correlation is significant at the 0.05 level (2-tailed)

**Correlation is significant at the 0.01 level (2-tailed)

## Discussion

Firstly, the upward trends in the occurrence and duration of floods revealed by this study could generally be deemed to have exacerbated the burden of malaria in five partner countries of FOCAC in E.A between 1990 and 2019. On the contrary, however, the result of inverse, negative, and weak correlations showed that malaria incidence in children < 5 years was not directly associated with flood occurrence and duration in five countries in the past three decades. Similar to this finding, some studies previously found climatic scenarios, in particular, floods, to have had either an inverse, negative, or neutral effect on malaria transmission in Ethiopia, Kenya, and Sudan [11, 12, 40]. The inverse, negative or weak correlations can, thus far, be attributed to the fact that our study did not take into account other factors or covariates, which are oftentimes associated with floods and have the potential to influence the risk and transmission of malaria in children < 5 years and other vulnerable groups. Among other socioeconomic factors may, include displacement and homelessness, food insecurity and malnutrition, disruption of healthcare services, and water, sanitation and hygiene (WASH). However, access, retrieval, and inclusion of data on these attributes for analysis of their potential influence on malaria incidence in children of < 5 years in five partner countries of FOCAC in E.A other than flood occurrence and duration was not possible in this study. This is because data in EM-DAT is not stratified or presents some information related to some of those socioeconomic factors.

Additionally, an inverse, negative, or weak correlation the flood occurrence and duration had with malaria incidence between 1990 and 2019 can be explained based on the fact that floods are preceded and triggered by excessive rainfall and water. This situation is inevitable in five countries and E.A region at large, given their tropical and coastal locations, and above all, vulnerability to climate change and its adverse effects [15, 41–43]. While rainfall vis-à-vis stagnant waters is the utmost factor reported to precipitate the risk of malaria [2, 44], they can wash off the mosquito breeding sites if they occur heavily or excessively. Also, heavy rainfall may destroy the development of mosquito eggs and larvae, or simply flash them out of the watersheds. To this matter, longer periods of flooding, which are reported to be frequent in the high- and low-lands of Ethiopia, Kenya, Somalia, and Sudan, are oftentimes characterized by excessive water runoff which definitely destroys and washes away mosquito breeding grounds and larvae [11, 12, 21, 23]. In the end, floods aggravated by excessive rainfall can positively help to reduce the risk of malaria, especially in children under-five years and other vulnerable groups of the population.

More so, the inverse relationship of floods with the burden of malaria in children < 5 years among five partner countries of FOCAC in E.A can be related to the impact of other natural hazards. They include drought, desertification, tropical storms, landslides, and mudslides, all of which are inextricably linked to or influenced by climate change [15, 41–43]. These climatic hazards, which often occur concurrently with floods in E.A region over the past decades, contribute to the spatial and temporal distribution of VBDs beyond malaria by influencing their interannual disparities and long-term trends. Apart from providing favorable grounds for mosquito breeding, growth, and survival [11, 12, 21, 23, 44], some of those natural hazards are associated with socioeconomic dynamics like population displacement, overcrowding, settlement, and appalling WASH conditions, which increase the odds of malaria transmission and other contagious diseases. It is undeniable that these conditions mostly affect children, as well as other vulnerable groups like women, elderly persons, and refugees, especially in the settings in Ethiopia, Somalia, and Sudan that have been plagued by armed conflicts, hunger, forced migration and protracted humanitarian crises [1, 7, 16, 28, 45].

Amid the continued burden of malaria in children < 5 years of age, the five E.A countries and many other less-developed alike are still having their healthcare systems grappling with various challenges —ranging from insufficient financing and shortage of health personnel to medical supplies [17, 46]. Moreover, malaria has been imposing not only a substantial burden of diseases on five E.A countries but also a threat to their socioeconomic growth and development, with a substantial GDP loss [5, 6, 47–49]. This has been particularly with the direct costs and expenditures on malaria prevention and treatment in terms of purchasing anti-malarial drugs, sprays, and bed nets [5]. In Tanzania, for instance, over 1.1% of its GDP represents US$2.2 per capita, and 39% of total national health expenditures were previously spent on malaria prevention and treatment [6]. These challenges, therefore, call for stakeholders, especially influential global actors to come to the rescue of five E.A and other malaria-endemic countries. Fortunately, China is one of the key development partners of E.A countries through the FOCAC initiative in which it made several substantial bilateral and multilateral commitments to assist Africa in areas like DRR, medical care, and public health [25, 26]. Through FOCAC, therefore, China can continue enhancing collaborations for anti-malaria projects involving pharmaceutical cooperation in producing medicines for malaria and other major infectious diseases [26]. Above all, China is committed to sharing its experience of anti-malaria successes with African countries following its recent malaria-free declaration by WHO in June 2021 [50]. No doubt, the fulfillment of these commitments would boost the malaria control programs so far undertaken, especially the innovation, distribution, and use of antimalarial medicines and mosquito nets [1, 2, 8, 9], which could have also contributed to a decline in malaria prevalence in the general population, including children among the five E.A countries.

By and large, cognizant of the devastating impacts of climate change and its associated effects, not just limited to floods only but also other natural hazards [18, 26, 51]; China also made a wide range of pledges for Africa to tackle the challenges of climate change. Notably, China is committed to helping African countries through the support of the Kunming Biodiversity Fund in promoting disaster prevention and relief, climate change, eco-environmental protection, and desertification prevention and treatment. Other promises related to promoting global environmental governance, strengthening exchanges and cooperation on ecological protection, developing the Great Green Wall, exchanging and cooperating on environmental industry and technology, and engaging in joint research on environmental issues [26]. It is undeniable that all these commitments, in one way or another, reinvigorate the efforts aimed at helping five E.A countries and the rest of Africa to attain the goal of malaria control and elimination and minimizing the risk of natural hazards by 2030 as stipulated in the Sustainable Development Goals (SDGs) [52] and Sendai Framework for Disaster Risk Reduction 2015–2030 (SFDRR)[53], respectively.

## Conclusions

The current study showed that the malaria incidence in children < 5 years in the past three decades was negative and weakly correlated with the increasing trends in floods among five partner countries of FOCAC in E.A. Possibly, this was due to the influence of different economic and sociocultural dynamics, as well as natural hazards that oftentimes compound floods, and, on the other hand, the concerted interventions taken to ameliorate the burden of malaria in five E.A countries. This, therefore, highlights the need for further studies to comprehensively explore how those factors or conditions, which oftentimes also complement floods, may influence the risk of malaria in children below five years in five E.A countries and elsewhere, where the disease is still endemic. This is highly warranted at this particular moment when the five partner countries of FOCAC and the E.A region at large are witnessing an increase in the occurrence of climate-induced floods and other extreme hazards. This ought to leverage the existing bilateral and multilateral partnerships, in particular, FOCAC —an initiative in which China is committed to addressing the different challenges faced by African countries, including the burden of malaria and climatic hazards like floods.

## Data Availability

The datasets generated and analyzed in this study are available from the corresponding author on reasonable request.

## References

[1] World Health Organization (WHO) (2022). World Malaria Report 2021.

[2] Ashley AE, Phyo AP, Woodrow JC, Malaria (2018). Lancet.

[3] Ngarakana-Gwasira ET, Bhunu CP, Masocha M. Mashonjowa E. *Assessing the Role of Climate Change in Malaria Transmission in Africa* Malaria Research and Treatment. 2016; 2016(7104291):7. 10.1155/2016/7104291 PMC4811621 27066290

[4] Achan J, Serwanga A, Wanzira H, Kyagulanyi T (2022). Current malaria infection, previous malaria exposure, and clinical profiles and outcomes of COVID-19 in a setting of high malaria transmission: an exploratory cohort study in Uganda. Lancet Microbe.

[5] Andrade VM, Noronha K, Diniz PCB, Guedes G (2022). The economic burden of malaria: a systematic review. Malar J.

[6] Jowett M, Miller NJ (2005). The financial burden of malaria in Tanzania: implications for future government policy. Int J Health Plann Manage.

[7] World Health Organization (WHO). The Africa Malaria Report 2003. 2003; WHO, *Geneva*.

[8] Nabarro DN, Tayler EM (1998). The Roll Back Malaria Campaign.

[9] Poore P, Nantulya V, Møgedal S, Okuonzi S. *The Global Fund to fight AIDS, tuberculosis and malaria (GFATM)*. Health Policy Plan. 2022; (19):52–3. 10.1093/heapol/czh006 14679285

[10] Patz JA, Epstein PR, Burke TA, Balbus JM (1996). Global climate change and emerging infectious diseases. JAMA.

[11] Kipruto EK, Ochieng AO, Anyona DN, Mbalanya M (2017). Effect of climatic variability on malaria trends in Baringo County, Kenya. Malar J.

[12] Sena L, Deressa W, Ali A (2015). Correlation of Climate Variability and Malaria: a retrospective comparative study, Southwest Ethiopia. Ethiop J Health Sci.

[13] Guha-Sapir D, Below R, Hoyois P (2021). Centre for research on the epidemiology of disasters (CRED). 2021 disasters in numbers:Extreme events defining our lives.

[14] Guha-Sapir D, Below R, Hoyois P (2021). Centre for research on the epidemiology of disasters (CRED). Annual Disaster statistical review 2021: the numbers and trends.

[15] Intergovernmental Panel on Climate Change (IPCC) (2018). Global warming of 1.5°C: an IPCC Special Report on the impacts of global warming of 1.5°C above pre-industrial levels and related global greenhouse gas emission pathways, in the context of strengthening the global response to the threat of climate change.

[16] Richardson K (2022). Climate risk report for the East Africa region.

[17] Suhr F, Steinert JI (2022). Epidemiology of floods in sub-saharan Africa: a systematic review of health outcomes. BMC Public Health.

[18] Balikuddembe KJ (2023). A Haddon matrix-based analysis of the anthropogenic drivers of floods in 10 eastern african partner countries of the Belt and Road Initiative 1990–2021. Int J Disaster Risk Reduct.

[19] World Health Organization (WHO). Vector borne diseases - key facts. 2017 [cited 2019 February 28]; Available from: https://www.who.int/en/news-room/fact-sheets/detail/vector-borne-diseases.

[20] Haileselassie W, Parker MD, Taye B, David ER (2022). Burden of malaria, impact of interventions and climate variability in western Ethiopia: an area with large irrigation based farming. BMC Public Health.

[21] Yan G, Githeko KA (2004). Association between climate variability and malaria epidemics in the east african highlands. PNAS.

[22] Jambou R, Njedanoun M, Panthou G, Descroix L (2022). Malaria Transmission in Sahelian African Regions, a Witness of Climate Changes. Int J Environ Res Public Health.

[23] Aal AR, Elshayeb AA (2011). The Effects of Climate Changes on the distribution and spread of Malaria in Sudan. Am J Environ Eng.

[24] Balikuddembe KJ (2022). Progressing Universal Health Coverage: Optimism or Pessimism for Vulnerable Population. J Disaster Emerg Res.

[25] Shelton G, Paruk F. *The Forum on China– Africa cooperation A strategic opportunity*, in *China International Studies*. Shelton G, Paruk F. Eds. 2008. Institute of Security Studies; 232.

[26] Forum on China-Africa Cooperation (FOCAC). Forum on China-Africa Cooperation Dakar Action Plan (2022–2024). 2021 [cited 2013 January, 2023]; Available from: http://www.focac.org/eng/zywx_1/zywj/202201/t20220124_10632444.htm.

[27] Balikuddembe KJ, Khorasani D, Ardalan A (2021). Risk factors associated with road traffic injuries at the prone-areas in Kampala city: a retrospective cross-sectional study. J Inj Violence Res.

[28] Spiegel PB, Le P, Ververs MT (2007). , *occurrence and overlap of natural disasters, complex emergencies and epidemics during the past decade (1995–2004)*. Confl Health.

[29] Nanjira D, Daniel DC. *Disasters and development in East Africa*, in *Managing natural disasters and the environment*. Alcira K, Mohan M. Eds. Biblioteca responsável: Washington D.C. 1990; 82 – 7.

[30] Douglas IK, Kurshid A, Campbell J (2008). Unjust waters: climate change, flooding and the urban poor in Africa. Environ and Urbanization.

[31] French Development Agency (AFD). Flood risk and cities in developing countries, in Technical Reports, Kovacs Y, Gaussens M, Eds. AFD. 2017: Paris.

[32] Li C, Yuan C, Lin Y, Hai L (2016). Spatio-temporal distribution of flood disasters and analysis of influencing factors in Africa. Nat Hazards.

[33] Guha-Sapir D, Below R, Hoyois P. *EM-DAT: The Emergency Events Database* in *The International Disaster Database– EM-DAT*, Centre for research on the epidemiology of disasters (CRED). Editor. 2022, Université catholique de Louvain (UCL) Brussels, Belgium.

[34] Integrated Research on Disaster Risk (IRDR). Peril classification and hazard glossary (IRDR data publication No.1) in Integrated Research on Disaster Risk. IRDR. 2014; Beijing.

[35] Institute for Health Metrics and Evaluation (IHME). *Global Burden of Disease*. Washington, USA.

[36] GBD 2019 Diseases and Injuries Collaborators (2020). Global burden of 369 diseases and injuries in 204 countries and territories, 1990–2019: a systematic analysis for the global burden of Disease Study 2019. Lancet.

[37] Abbafati C, Abbas KM, Abbasi-Kangevari M (2020). Global burden of 369 diseases and injuries in 204 countries and territories, 1990 – 2019: a systematic analysis for the global burden of Disease Study 2019. Lancet.

[38] World Health Organization (WHO). Classification of Diseases (ICD). WHO. 2011; Geneva.

[39] R Core Team. R: A language and environment for statistical computing. 2021 [cited 2023; Available from: https://www.R-project.org/.

[40] Hussien HH (2019). Malaria’s association with climatic variables and an epidemic early warning system using historical data from Gezira State, Sudan. Heliyon.

[41] Intergovernmental Panel on Climate Change (IPCC). Climate Change 2014: Synthesis Report. Contribution of Working Groups I, II and III to the Fifth Assessment Report of the Intergovernmental Panel on Climate Change. IPCC. 2015; Geneva.

[42] Tollesfson J (2021). IPCC climate report: Earth is warmer than it’s been in 125,000 years. Nature.

[43] Serdeczny O, Adams S, Baarsch F, Coumou D (2015). Climate change impacts in Sub-Saharan Africa: from physical changes to their social repercussions. Reg Environ Change.

[44] Ding G, Gao L, Li X, Zhou M (2014). A mixed method to evaluate Burden of Malaria due to flooding and waterlogging in Mengcheng County, China: a Case Study. PLoS ONE.

[45] Ahmed A, Mulatu K, Elfu B (2021). Prevalence of malaria and associated factors among under-five children in Sherkole refugee camp, Benishangul-Gumuz region, Ethiopia. A cross-sectional study. PLoS ONE.

[46] Balikuddembe JK, Fu B, Reinhardt DJ. *Healthcare Challenges after Disasters in Lesser developed Countries*. Oxf Res Encyclopedia Nat Hazard Sci, 2019:1–30.

[47] United Nations Development Programme (UNDP). Baseline study on disaster recovery in Africa, in Transitioning from relief to recovery. Kambon A, et.al. Eds. UNDP. 2019; New York.

[48] World Bank. Flood Exposure and Poverty in 189 Countries, in Poverty and Shared Prosperity 2020. Rentschler J, et.al. Eds. World Bank. 2020; Washington D.C.

[49] Nanjira D et al. *Disasters and development in East Africa*, in *Managing natural disasters and the environment*. Alcira K, et.al. Eds. Biblioteca responsável. 1990; 82 – 7, Washington.

[50] Wang D, Lv S, Ding W et al. *Could China’s journey of malaria elimination extend to Africa?* Infect Dis Poverty. 2022; 11(55). 10.1186/s40249-022-00978-w PMC9108373 35578325

[51] Balikuddembe JK, Baofong D, Ruixin Z, Shaolin W (2021). A multisource trend analysis of floods in Asia-Pacific 1990–2018: implications for climate change in sustainable development goals. Inter J Disaster Risk Reduction.

[52] United Nations (UN). Transforming our world: the 2030 agenda for sustainable development. UN. 2015; New York.

[53] United Nations International Strategy for Disaster Reduction (UNISDR). *Sendai framework for disaster risk reduction 2015–2030*. UNISDR. 2015; Geneva.

